# Evaluation of antibacterial activity and chemical analysis of clove aqueous extract (*Syzygium aromaticum*)

**DOI:** 10.1186/s12906-023-04243-x

**Published:** 2025-04-21

**Authors:** Mahasin Ahmed Wadi

**Affiliations:** Department of Medical-Surgical, College of Nursing, Princess Nourah Bint Abdulrahman University, P.O. Box 84428, Riyadh, 11671 Saudi Arabia

**Keywords:** Antibacterial activity, Standard organisms, Chemical analysis, Clove aqueous extract (*Syzygium aromaticum*)

## Abstract

Clove (*Syzgium aromaticum*) is an aromatic historic spice from the Myrtaceae family. The clove’s major chemical ingredients are essential oils.

Cloves have long been utilized in both traditional and modern medicine. The Myrtaceae family’s clove bud (*Syzygium aromaticum)* is regarded as one of the most efficient and important antibacterial natural herbs.

The aim of the present study was to evaluate the antibacterial properties of clove aqueous extracts (*Syzygium aromaticum*) and its chemical characterization. To verify the nature of the antibacterial agent (s) of clove.

Clove aqueous extract was evaluated for antibacterial activity in vitro against 10 Gram-positive and Gram-negative standard organisms using well diffusion method, *Campylobacter coli* (*C. coli*): ATCC:43478, ATCC: *Enterobacter aerogenes* (*E*. *aerogenes*): ATCC: 13048, *Enterococcus faecalis* (*E*. *faecalis*): ATCC: 29212, *Escherichia coli* (*E*. *coli*): ATCC: 25922. *Klebsiella pneumoniae* (*K. pneumoniae*): ATCC: 700603, *Listeria monocytogenes* (*L*. *monocytogenes*): ATCC 35152, *Pseudomonas aeruginosa* (*P*. *aeruginosa*): ATCC: 27853, *Shigella sonnei* (*S*. *sonnei*): ATCC12022, *Staphylococcus aureus *(*S. aureus*): ATCC: 29213, and *Staphylococcus Methicillin* Resistant MRSA: ATCC: 2359.

Clove ethyl acetate extract was subjected to Gas chromatography-Mass spectrometer GC–MS for chemical characterization.

Clove aqueous extract exerted a potent antimicrobial activity against the 10 standard organisms.

Substantial broad spectrum antibacterial activity was reported in clove aqueous extract against 10 common Gram-positive and Gram-negative bacteria. *E. coli* and *K. pneumoniae* were found to be the most sensitive bacteria to the clove aqueous extract. Clove’s chemical makeup was identified using GC–MS. 58 different chemicals in total were found. Eugenol has the highest concentration (53.24).

## Introduction

Cloves are used in Ayurveda, Chinese medicine and Western herbalism. Essential oils and their constituents are being used in modern medicine for their medicinal effects. Many volatile chemicals are actually common components in pharmaceuticals preparations. People who are suffering from the adverse affects of antimicrobial resistance are turning to natural products to find relief. Herbs are utilized to treat a variety of infectious diseases all over the world, and medicinal herbs can provide a plethora of antibacterial compounds. Herbal medicines are plant-derived compounds with medicinal qualities that are advantageous to human health [1]. When compared to sodium benzoate, potassium sorbate, and other chemical food preservatives, clove oil, the major volatile ingredient of *S. aromaticum*, exhibits a number of benefits in terms of antibacterial activity, fragrances, and safety, and is an excellent alternative to chemical food preservatives [2].

Cloves (*Syzygium aromaticum*, known as Eugenia aromaticum or Eugenia caryophyllata) are the fragrant dried flower buds of a *Myrtaceae* tree [3]. It’s also applied in dentistry, where clove essential oil is used as an anadyne in case of a dental emergency [4]. Clove oil is also applied in the fragrance and flavoring industries, and is used as a topical application to reduce pain and promote healing [5]. Clove and cinnamon essential oils have emerged as efficient antibacterial agents, as indicated by inhibition zone diameter and the minimum inhibitory concentration (MIC) value against extended-spectrum βlactamase ESBL-producing *E. coli* and *K. pneumoniae* isolates [6]. Essential oils of *Syzygium aromaticum* (clove) and *Cinnamomum verum* (*cinnamon*) have the most antibacterial components, eugenol in clove and cinnamaldehyde in cinnamon, both of which exhibit antibacterial activity against foodborne pathogens [7]. Clove extract exhibited antibacterial activity against all tested Gram-negative uropathogens [8]. The ethanolic extract of clove (260.5 mm) provided the largest zone of inhibition against *K. pneumonia*, followed by the zones of inhibition produced by ethanolic extract of clove against MRSA (20 mm) at 1.0 g/mL concentration [9]. Clove (*Syzygium aromaticum*) exhibited antibacterial activity against pathogenic bacteria isolated from fish culture [10]. The produced clove oil had good antibacterial activity against *S. aureus* and *E.coli* and was non-cytotoxic to human fibroblast cell lines. It also had good wound-healing capability [11]. Clove essential oil (CEO) was reported to possess antibacterial, antifungal, insecticidal, and antioxidant properties [12]. Thymol is recommended in dentistry because of its antibacterial characteristics, while eugenol is advised because of its analgesic properties [13].

Previous studies have shown that the essential oil from clove buds has antibacterial activity against a variety of food-borne microorganisms [14]. Natural products, as substitutes of synthetic chemical preservatives, are increasingly accepted because they are innately better tolerated in human body and have inherent superiorities for food industry [15]. Clove essential oil, derived from dried flower buds, is used topically to treat pain and promote healing, as well as in the pharmaceutical, fragrance, and flavoring industries [5]. Clove essential oil has a variety of pharmacological and biological properties, including antioxidant activity [16].

For grounded and ungrounded seeds, GC–MS analysis revealed the presence of 17 heterogeneous chemicals, including eugenol (68.7–87.4%), cyperene (20.5–7.2%), phenethyl isovalerate (6.4–3.6%), and cis-thujopsene (1.9–0.8%) [17]. The strongest bactericidal activity of eugenol on *E. coli* is detected when the bacteria is exposed to eugenol for the first 10 min [18]. Eugenol has antimicrobial activity against *S. agalactiae* planktonic cells, and this activity is time-dependent as shown by viability tests and time–kill curves [19]. *S. aureus* and *S. epidermidis* can be significantly inhibited by eugenol [20]. Eugenol (0.01% V/V) inhibits the swarming motility and hemolytic activity of *P. aeruginosa* and reduces the formation of pyocyanin and 2-heptyl-3-hydroxy-4(1H)-quinolone [21]. Eugenol microemulsion can prolong the stagnation period of *L. monocytogenes* in whole-fat milk [21]. Clove oil and eugenol showed superior inhibition effect against *B. cereus* than they do against *E. coli*, *Salmonella*, *P.aeruginosa*, and *L. monocytogenes* [22]. Only four components are found when clove buds are extracted using steam distillation and GC–MS: 3-allyl-2methoxyphenol (69.77 percent), 3-phenyl-2-propen aldehyde (14.32 percent), caryophyllene (13.74 percent), and alpha-Caryophyllene (2.17 percent) [23]. Essential oils (EOs) are complex combinations of aromatic plants’ bioactive compounds. EOs are liquid, soluble in organic solvents and soluble in lipids, some of them are colorless and others range from a light yellow to a reddish orange, such as lemongrass oil, cinnamon oil, and sandal oil; mainly, EOs are less dense than water, such as citronella oil, lime oil or orange oil, but there are some heavier than water, such as allspice oil, cinnamon oil, clove oil or garlic oil [24]. According to several sources, *S. aromaticum* contains 15–20% EO by weight. CEO is high in phenolic compounds, which have a variety of biological actions such as antibacterial, antifungal, insecticidal, and antioxidant characteristics [25]. Propolis essential oil has excellent promise for both the prevention and treatment of oral bacterial infections induced on by Streptococcus mutans [26]. Propolis essential oil has the potential to be developed into a medication to prevent and treat dental caries brought on by Streptococcus mutans due to its strong antibacterial and antibiofilm activity against *Streptococcus* mutans [27].

The anti-biofilm characteristics of essential oils in the prevention, eradication, and control of bacterial biofilm dispersion on surfaces in contact with food [28].

The aim of the current research is to assess the chemical profile and antibacterial efficacy of clove aqueous extracts (*Syzygium aromaticum*) against 10 standard organisms. To verify the nature of clove’s antibacterial agent(s).

## Materials and methods

### Plant material and extraction

Commercial food-grade clove buds (*Syzygium aromaticum*), were purchased from local market at Saudia Arabia during April 2022. Cloves was stored in an airtight container, in a cool and dark place.

About 25 g of clove was soaked in 25 ml sterile distilled water for over night, and left for 24 h at room temperature with occasional shaking and filtered to obtain 50% clear aqueous extract. A sterile glass container has been used to store clove solution. The aqueous extract was held in a refrigerator (4 °C) until the analysis was performed. A 25 gm of clove bud sample was soaked with 25 ml sterile distilled water to give 50% dilution ( clove aqueous extract). The diluted clove sample was extracted with 50 ml (5X10 ml) of ethyl acetate using liquid/liquid extraction technique, using separating funnels. Ethyl acetate was used to demonstrate the volatile components of clove using the.

Gas Chromatography Mass Spectrometer. Anhydrous sodium sulphate was used to separate the top organic phase (ethyl acetate), which was concentrated to 10 ml at reduced pressure [29].

The clove aqueous extract was collected and examined for sterility at the microbiology laboratory.

### Chemicals and reagent

All chemicals and reagents were analytically grade purity.

### Standard organisms

The following 10 standard organisms Gram-positive, Gram -negative, Microbiology Reference Laboratories were obtained, the American Type Culture Collection (ATCC), 12301 Drive, Rock Ville, MD 20852, and USA. *Campylobacter coli* (*C. coli*): ATCC:43478, ATCC: *Enterobacter aerogenes* (*E*. *aerogenes*): ATCC: 13048, *Enterococcus faecalis* (*E*. *faecalis*): ATCC: 29212, *Escherichia coli* (*E*. *coli*): ATCC: 25922. *Klebsiella pneumoniae* (*K. pneumoniae*): ATCC: 700603, *Listeria monocytogenes* (*L*. *monocytogenes*): ATCC 35152, *Pseudomonas aeruginosa* (*P*. *aeruginosa*): ATCC: 27853, *Shigella sonnei* (*S*. *sonnei*): ATCC12022, *Staphylococcus aureus*(*S. aureus*): ATCC: 29213, and *Staphylococcus Methicillin* Resistant MRSA: ATCC: 23591 [29].

### Inoculum preparation

Pure culture and standard inoculum size has been maintained for antibacterial susceptibility. Control organisms were suspended in a sterile saline to match 0.5 McFarland standard tube, which is commercially available, provide an optical density of 1.5 X 10^8^ Colony forming units (CFU/ml). The bactericidal activity of clove aqueous extract was tested using the well plate technique [30].

### Well plate technique

The seeded agar diffusion technique was used [26]. Muller Hinton agar culture medium was reconstituted and sterilized (using an autoclave) at 121 °C for 15 min, then cooled at 48 °C before being inoculated with 0.1 ml of standardized 24 broth culture of bacterial suspensions that match the turbidity of the 0.5 McFarland standard tube (1.5X10^8^) (FU/ml). Standard conditions for antimicrobial susceptibility testing procedures have been proposed based on several laboratory experiments. Guidelines and recommendations for their use are published by the National Committee for clinical laboratory standards (NCCLS), NCCLS, 940 W. valley Road, suite 1400, Wayne, pa.. 19087. The inoculated medium was poured onto sterile Petri-dishes with internal diameters of 95 mm and allowed to set aseptically in 20 ml volumes. The solidified seeded agar plate was then kept at 4 °C until usage. Four cups (8 mm diameter) were cut using 8 mm sterile cork borer, and the cut-disc of agar was removed 0.2 ml of each honey sample was carefully added to diffuse. The seeded plates were incubated at 37 °C for 18–24 h [29]. The diameter of the resultant growth inhibition zone was measured in (mm) in mm ± standard deviation (SD). In four replicates, clove aqueous extract was evaluated for antibacterial properties against each organism. The average diameter of the inhibition zone was measured. The standard deviation was estimated.

### In vitro antibacterial activity of Clove aqueous extract

Using the well plate technique, a clove aqueous sample was tested in four duplicates against 10 standard bacteria. The average diameter of the inhibitory zone was calculated.

### Gas Chromatography Mass Spectrometer (GC- MS) preparation

The Clove aqueous sample was analyzed with a GC–MS SHIMADZUQP5050 GC-174 equipped with an electron impact detector and a column RTX5M5 packed with 5% diphenyl-95 percent diethyl polysiloxane. The gas carrier was Helium, and the length was 30 m, the interior diameter was 0.25 mm, and the film thickness was 0.25 mm. The injector and detector temperatures were kept at 50 °C until they reached 280 °C, respectively [31].

Clove aqueous extract sample was injected automatically. Electron impact mass spectra in the 40–500 mass range were observed. 70 V of electron ionization energy 2 min to start, 45 min to finish During the elution process, software was employed to automatically record spectral data. Beak development was compared to a mass spectra database for identification [27].

### Statistical analysis

All the analysis were carried out in triplicate and the experimental results obtained were expressed as means ± standard deviation.

## Results

### In vitro antibacterial activity of clove aqueous extract

The antibacterial activity of a clove aqueous extract sample was tested against ten standard bacterial strains, Gram-positive and Gram-negative, *C. coli*: ATCC:43,478, *E. aerogenes*:ATCC:13,048, *E. faecalis*: ATCC:29,212, *E.coli*: ATCC: 25,922. *K*. *pneumoniae*: ATCC: 700,603, *L. monocytogenes*: ATCC 35152, *P*. *aeruginosa*: ATCC: 27,853, *S*. *sonnei*, *S*. *aureus*: ATCC: 29,213,: ATCC: 12,228, and MRSA: ATCC: 23,591. Clove aqueous extract was found to have potent antimicrobial activity against all tested Gram-positive and Gram-negative bacteria. Different antibacterial activity was demonstrated by Clove aqueous extract against all Gram-negative. *E*. *coli* exhibited the highest susceptivity to the Clove aqueous extract 33 ± (1) mm.

Followed by *K*. *pneumoniae* 32 ± (1) mm Table 1. The growth inhibition zone of clove aqueous extract suggested that it has inhibitory effects on both Gram-positive and Gram-negative organisms Table 1. *S. aureus* 28 ± (0.5) mm, *S. aureus*, Meithicillin Resistant MRSA29 ± (0.5) mm, were found to be the most sensitive Gram-positive organisms to the clove aqueous extract Table 1. Although *K. pneumoniae* 32 ± (1)mm, *E*. *aerogenes* 32 ± (1)mm,* S*. *sonnei* 28 ± (0.5) mm. and *L*. *monocytogenes* 27 ± (0.8) mm. showed marked sensitivity to the tested clove aqueous extract sample. The Gram-negative bacteria *P*. *aeruginosa*, which is most resistant to widely used antibiotics, was found to be susceptible to the tested clove aqueous extract 27 ± (0.8) mm Table 1. In terms of magnitude, Gram-positive and negative bacterial showed nearly comparable inhibitory zones.

**Table 1:**
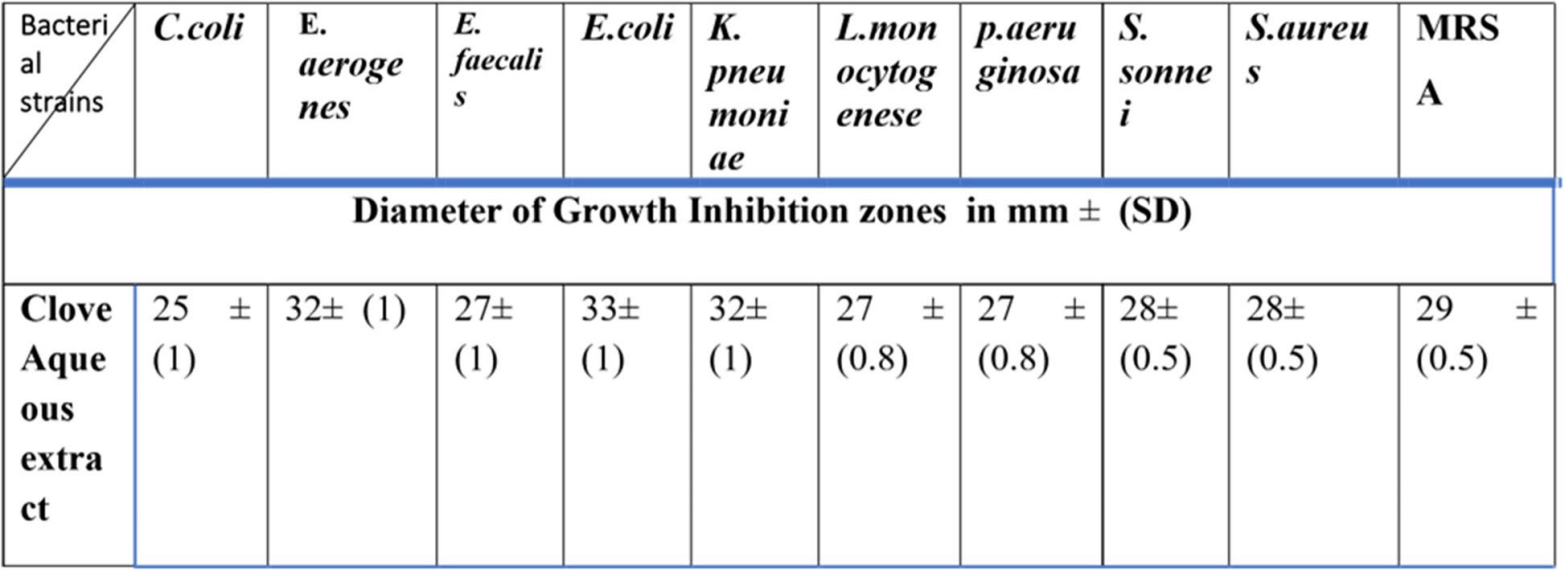
Antibacterial activity of clove aqueous extract against 10 standards organisms

### Gas Chromatography Mass Spectrometer (GC–MS) analysis of clove ethyl acetate extract

#### GC–MS chromatogram of clove ethyl acetate extract

Mass spectrometry (MS), which offers the crucial data to define component structure, and capillary column gas chromatography (GC), which separates mixture components, are combined to create the GC–MS analytical approach.

The chromatograms are interpreted with the help of the “Data Analysis” application. Short retention time peaks were predominantly volatile oxygenated chemicals, whereas long retention time peaks were semi volatile molecules.

**Fig. 1:**
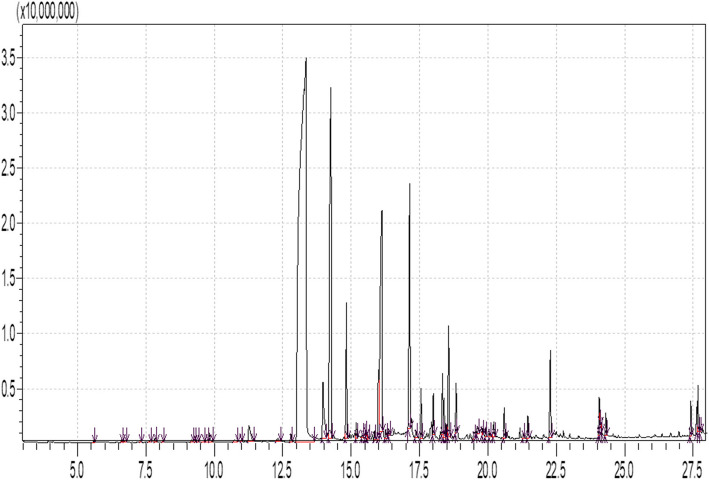
Eluted components of clove ethyl acetate extract

The sample’s components were identified by comparing their retention durations and mass fragmentation patents to those in the National Library Institute of Standards and Technology’s database (NIST). Gas Chromatography Mass Spectrometer (GC–MS) analysis of a clove aqueous extract detected 58 compounds Table 2. Eugenol has the highest concentration, (53.24) major peaks were identified as eugenol Table 2, Fig. 1. The major identified component was eugenol. Followed by Caryophyllene (11.61), Phenol, 2-methoxy-4-(2-propenyl) -, acetate (8.22), Caryophyllene oxide (5.38), Vanillin (1.92). A number of phenolic derivatives was identified 4-(2-propenyl)-(0.77), Phenol, 4-[2,3-dihydro-7-methoxy-3-methyl-5-(1propenyl)-2-benzofuranyl]-2-methoxy-(0.68) Table 2.

**Table 2:** Identification of chemical compounds of clove ethyl acetate extract

ID#	Name	Ret.Time	Area	Area%
1	5-Hepten-2-one, 6-methyl-	5.587	350973	0.03
2	Acetic acid, 5-methylhex-2-yl ester	6.600	394622	0.04
3	Benzyl alcohol	6.689	506485	0.05
4	.alpha.-Methyl-.alpha.-[4-methyl-3pentenyl]oxiranemethanol	7.284	148487	0.01
5	2-Nonanone	7.631	467639	0.04
6	1,6-Octadien-3-ol, 3,7-dimethyl-	7.824	696941	0.06
7	Geranyl nitrile	8.104	236862	0.02
8	Acetic acid, phenylmethyl ester	9.165	630479	0.06
9	Benzoic acid, ethyl ester	9.300	133674	0.01
10	2H-Pyran-3-ol, 6-ethenyltetrahydro-2,2,6trimethyl-	9.393	48693	0.00
11	Octanoic acid	9.547	128951	0.01
12	Benzoic acid	9.746	1092019	0.10
13	Methyl salicylate	9.821	2299047	0.21
14	Benzofuran, 2,3-dihydro-	10.737	1376725	0.13
15	Geraniol	10.977	57901	0.01
16	Phenol, 4-(2-propenyl)-	11.268	8436365	0.77
17	2-Methoxy-4-vinylphenol	12.320	1461343	0.13
18	.alpha.-Cubebene	12.794	1395164	0.13
19	Eugenol	13.353	585790880	53.24
20	Vanillin	13.971	21136137	1.92
21	Caryophyllene	14.258	127746324	11.61
22	1,4,7,-Cycloundecatriene, 1,5,9,9-tetramethyl-, Z,Z,Z-	14.827	27646457	2.51
23	.gamma.-Muurolene	15.184	2687243	0.24
24	Naphthalene, decahydro-4a-methyl-1methylene-7-(1-methylethenyl)-, [4aR(4a.alpha.,7.alpha.,8a.beta.)]-	15.400	580833	0.05
25	.alfa.-Copaene	15.509	554012	0.05
26	Naphthalene, 1,2,3,4,4a,5,6,8a-octahydro-4a,8dimethyl-2-(1-methylethenyl)-, [2R(2.alpha.,4a.alpha.,8a.beta.)]-	15.548	458589	0.04
27	.alpha.-Muurolene	15.595	1017471	0.09
28	1H-Cyclopropa[a]naphthalene, 1a,2,3,5,6,7,7a,7b-octahydro-1,1,7,7atetramethyl-, [1aR-(1a.alpha.,7.alpha.,7a.alpha.,7b.alpha.)	15.856	1645379	0.15
29	Naphthalene, 1,2,3,5,6,8a-hexahydro-4,7dimethyl-1-(1-methylethyl)-, (1S-cis)-	15.998	16163116	1.47
30	Phenol, 2-methoxy-4-(2-propenyl)-, acetate	16.120	90477615	8.22
31	2-Propanone, 1-(4-hydroxy-3-methoxyphenyl)-	16.261	2377744	0.22
32	.alpha.-Calacorene	16.377	1209900	0.11
33	Caryophyllene oxide	17.139	59218343	5.38
34	Humulene	17.369	1701187	0.15
35	2,5,9-Trimethylcycloundeca-4,8-dienone	17.551	9155163	0.83
36	Alloaromadendrene oxide-(1)	18.005	7465532	0.68
37	Androstan-17-one, 3-ethyl-3-hydroxy-, (5.alpha.)-	18.337	11769787	1.07
38	.beta.-iso-Methyl ionone	18.391	7700013	0.70
39	(7a-Isopropenyl-4,5-dimethyloctahydroinden-4yl)methanol	18.490	2508018	0.23
40	1H-Cycloprop[e]azulen-4-ol, decahydro-1,1,4,7tetramethyl-, [1aR-(1a.alpha.,4.beta.,4a.beta.,7.alpha.,7a.beta.,7b.alp ha.)]-	18.564	22927451	2.08
41	2',3',4' Trimethoxyacetophenone	18.832	9258607	0.84
42	Benzeneacetic acid, 4-hydroxy-3-methoxy-, methyl ester	19.494	2614031	0.24
43	4-Hydroxy-2-methoxycinnamaldehyde	19.616	2679209	0.24
44	Tetradecanoic acid	19.791	1148645	0.10
45	Benzyl Benzoate	19.923	1747868	0.16
46	Cyclopenta[cd]pentalen-2-one, 4,4,6a,6btetramethylhexahydro-1-oxa-2a,3-diaza-	20.070	1043187	0.09
47	Ledol	20.212	3464204	0.31
48	4aH-cycloprop[e]azulen-4a-ol, decahydro-1,1,4,7-tetramethyl-	20.595	5297996	0.48
49	Benzoic acid, 2-hydroxy-, phenylmethyl ester	21.298	975407	0.09
50	4,4,8-Trimethyltricyclo[6.3.1.0(1,5)]dodecane-2,9-diol	21.461	4502033	0.41
51	n-Hexadecanoic acid	22.279	19364260	1.76
52	9,12-Octadecadienoic acid (Z,Z)-	24.070	6596451	0.60
53	Oleic Acid	24.101	1052067	0.10
54	6-Butyl-1,4-cycloheptadiene	24.138	856938	0.08
55	Octadecanoic acid	24.306	4005601	0.36
56	[1,1'-Biphenyl]-2,2'-diol, 3,3'-dimethoxy-5,5'-di-2-propenyl-	27.415	5891444	0.54
57	Phenol, 4-[2,3-dihydro-7-methoxy-3-methyl-5(1-propenyl)-2-benzofuranyl]-2-methoxy-	27.674	7536827	0.68
58	Bikaverin	27.756	725298	0.07

While, mostly minor constituents contains Acetic acid, 5-methylhex-2-yl ester (0.04), Acetic acid, phenylmethyl ester (0.06), Benzoic acid (0.10), Benzeneacetic acid, 4-hydroxy-3-methoxy-, methyl ester(0.24), nHexadecanoic acid (1.76), Oleic Acid (0.10), Octadecanoic acid (0.36), 9,12Octadecadienoic acid (Z,Z)- (0.60), Table 2.

## Discussion

Clove bud (*Syzygium aromaticum*) is one of the most efficient and significant antibacterial natural plants. Eugenol is the main component of clove oil. The primary volatile component of *S. aromaticum*, clove oil, exhibits a variety of benefits in terms of antibacterial activity, fragrances, and safety, making it a perfect alternative to synthetic food preservatives. Foodborne illnesses like campylobacteriosis are a major global public health problem [32]. The findings of the present study showed that *E. coli* demonstrated a marked sensitivity towards tested clove aqueous extract (33 mm). The obtained results is consistent with the previous findings that eugenol expresses higher antimicrobial efficacy on *E. coli* K12 [33]. *E. coli* in the medical field is becoming more and more of a resistant threat [34].* K*. *pneumoniae* showed remarked inhibitory effects toward clove aqueous extract. It’s already been demonstrated the same observation by the previous findings [8, 9]. Our current results showed that the clove aqueous extract is effective against *P*. *aeruginosa*, which is the most resistant to common antibiotics [35]. Eugenol (0.01%, V/V) can suppress *P. aeruginosa* swarming motility and hemolytic activity as well as decrease the formation of pyocyanin and 2-heptyl-3-hydroxy-4(1H)-quinolone [35].The findings of the present study revealed that *S. sonnei* is sensitive to clove aqueous extract.

The present study noted that *S. aureus*, Meithicillin Resistant MRSA, was found to be the most sensitive Gram-positive organisms to the tested clove aqueous extract. *S. aureus* can be significantly inhibited by eugenol [20]. Eugenol can also destroy the biofilm integrity and reduce the adhesion of methicillin-resistant *S. aureus* (MRSA) [20].

The findings of the current study confirmed that *L. monocytogens* was found to be susceptible to clove aqueous extract. The *L. monocytogenes* stagnation period in wholefat milk can be prolonged by eugenol microemulsion [30]. Furthermore, clove has been proven successfully against *Listeria monocytogenes* in food systems [36]. Similarly, in another study clove oil was found active against foodborne Gram positive bacteria (*S. aureus*, *B. cereus*, *E. faecalis* and *L*. *monocytogenes*) and Gram-negative bacteria (*E. coli*, *Yersinia enterocolitica*, *Salmonella choleraesuis* and *P. aeruginosa*) [37]. Clove’s main ingredient had already been associated to its major element eugenol [31].

The antibacterial activity of eugenol against *S. typhi* is due to the interaction of eugenol on bacterial cell membrane [38]. Alternatively, a variety of dental care products containing clove oil have entered the Chinese market [2].

The present study noted that clove extract detected 58 compounds Table 2. Eugenol has the highest concentration, (53.24) major peaks were identified as eugenol, and is known to possess antimicrobial activity against many pathogens [28]. Similar findings confirmed the presence of 17 heterogeneous compounds, including eugenol (68.7–87.4%), cyperene (20.5–7.2%), phenethyl isovalerate (6.4–3.6%), and cis-thujopsene, was detected in both grounded and ungrounded seeds by GC–MS analysis (1.9–0.8%) [17].

Eugenol was found to be the major component of the clove aqueous extract 53.24%. It has been previously reported that Eugenol (75.30%), Eugenyl Acetate (20.93%), and -caryophyllene are the three main substances in the bud oil (3.00 percent) [39, 40]. Eugenol is a phenolic compound. In accordance with earlier observations that phenols are known to have antibacterial characteristics [28]. This is supported by antibacterial evidence acquired for these compounds. Phenolic compounds are known to have antioxidant and antimicrobial properties [29].

The first investigation found that the phenolic extract of cloves (*Syzygium aromaticum*) possesses antibacterial properties against the growth of *S. aureus* and *E. coli* at a concentration of 100 mg/mL [30]. The potent biological and antibacterial properties of clove essential oil are due to the high amounts of eugenol it contains. It is well known that the phenolic chemicals in clove essential oil and eugenol can denature proteins, interact with phospholipids in cell membranes to change how permeable they are, and inhibit a wide range of Gram-negative and Gram-positive bacteria [34].

## Conclusion

Clove aqueous extract displayed marked broad spectrum antibacterial activity against 10 standard Gram-positive and Gram-negative bacteria. The most susceptible bacteria to the clove aqueous extract were *E. coli* and *K. pneumoniae*. Chemical composition of clove was determined by GC–MS. A total of 58 compounds were detected. Main components in the Clove are eugenol. Eugenol is the main component of clove (53.24). Since clove includes a lot of potent ingredients and has a strong aroma, it has been used as a condiment in traditional Chinese foods for more than 2000 years. The antibacterial properties of clove bud oil are due to the phytochemicals contained.

One of the phytoconstituents that may have greatly aided the antibacterial activities is eugenol. So, clove as natural product may be a useful adjuvant, especially in the treatment against certain pathogens. Herbal medicine is currently gaining popularity as a secure and reliable method of treating a wide range of medical issues. Cloves may be used as efficient all-natural treatments for a variety of foodborne illnesses. It is necessary to conduct additional research on the safety and effectiveness of such substances to see whether they can provide therapeutic benefits either on their own or in conjunction with traditional medicines.

## Data Availability

All data and materials were included in the manuscript.

## Abbreviations

GC- MS: Gas Chromatography Mass Spectrometer
*S. aromaticum*: *Syzgium aromaticum*
*E*. *aerogenes*: *Enterobacter aerogenes*
*E*. *faecalis*: *Enterococcus faecalis*
*E*. *coli*: *Escherichia coli*
*K. pneumoniae*: *Klebsiella pneumoniae*
*L*. *monocytogenes*: *Listeria monocytogenes*
*P*. *aeruginosa*: *Pseudomonas aeruginosa*
*S*. *sonnei*: *Shigella sonnei*
*S. aureus*: *Staphylococcus aureus*
MRSA: *Staphylococcus Methicillin* Resistant
